# Supplementary material for: EnzML: multi-label prediction of enzyme classes using InterPro signatures
Source: BMC Bioinformatics. 2012 Apr 25;13:61. doi: 10.1186/1471-2105-13-61 (PMC3483700; doi:10.1186/1471-2105-13-61)
Supplement: Addtional file 5 — The Java code to format the data files, evaluate and predict. The file enzml_java_code.tar.gz contains the Java code used to format database data to ARFF and XML formats, to execute cross and train-test (jackknife) evaluations and to record evaluation results to database. More information is included in the readme.txt file and the Javadoc files. The code can be used with a MySQL database. To use a different database software, other JDBC drivers might be required. [file 1471-2105-13-61-S5.gz › java_code/enzml2011/doc/uk/ac/ed/inf/taweka/mulan/predict/MulanPredict.html]

MulanPredict


---


|  |  |  |
| --- | --- | --- |
| **Overview** \| **Package** \| **Class** \| **Use** \| **Tree** \| **Deprecated** \| **Index** \| **Help** | |  |
| PREV CLASS   **NEXT CLASS** | **FRAMES**    **NO FRAMES**     **All Classes** |  |
| SUMMARY: NESTED \| FIELD \| CONSTR \| METHOD | DETAIL: FIELD \| CONSTR \| METHOD |  |


---


## uk.ac.ed.inf.taweka.mulan.predict Class MulanPredict

```
java.lang.Object
  uk.ac.ed.inf.taweka.Initialised
      uk.ac.ed.inf.taweka.mulan.predict.MulanPredict
```

**All Implemented Interfaces:**: Initialisable

**Direct Known Subclasses:**: MulanPredictWithTrainedModel

---

``` public class MulanPredict extends Initialised ```

First trains on a dataset (training set) and then emits predictions for an
unlabelled dataset.

**Version:**
:   19 Sep 2010

**Author:**
:   Luna De Ferrari luna.deferrari-at-ed.ac.uk

---

| **Field Summary** | |
| --- | --- |
| `static java.lang.String` | `ATTRIBUTE_ID` |
| `static java.lang.String` | `ATTRIBUTE_NAME` |
| `static java.lang.String` | `BIPARTITION` |
| `static java.lang.String` | `CONFIDENCE` |
| `static java.lang.String` | `INSTANCE_ID` |
| `static java.lang.String` | `INSTANCE_NAME` |
| `static java.lang.String` | `RANKING` |

**Fields inherited from class uk.ac.ed.inf.taweka.Initialised**

`m_initialisationIsCorrect`


**Constructor Summary**

`MulanPredict(int trainArffId,
int unlabeledArffId,
java.lang.String arffRecordsDatabase,
java.lang.String predictionsDirectory)`


| **Method Summary** | |
| --- | --- |
| `java.util.Vector<java.lang.String>` | `generateInstancesNames()` |
| `java.lang.String` | `getLogFile()` |
| `MulanArffRecord` | `getTrainArff()` |
| `MulanArffRecord` | `getUnlabeledArff()` |
| `static void` | `main(java.lang.String[] args)` |
| `void` | `predict()` |
| `static java.lang.String` | `predictionsHeader()` |
| `java.lang.String` | `processOutput(int instanceIndex, mulan.classifier.MultiLabelOutput instanceOutput)` |
| `void` | `savePredictions()` |
| `void` | `setPredictionsFile(java.lang.String predictionsDirectory)` |

**Methods inherited from class uk.ac.ed.inf.taweka.Initialised**

`initialisationIsCorrect, setInitialisationIsCorrect`

**Methods inherited from class java.lang.Object**

`equals, getClass, hashCode, notify, notifyAll, toString, wait, wait, wait`

**Field Detail**

### INSTANCE\_ID

```
public static final java.lang.String INSTANCE_ID
```

**See Also:**: Constant Field Values

---


### INSTANCE\_NAME

```
public static final java.lang.String INSTANCE_NAME
```

**See Also:**: Constant Field Values

---


### ATTRIBUTE\_ID

```
public static final java.lang.String ATTRIBUTE_ID
```

**See Also:**: Constant Field Values

---


### ATTRIBUTE\_NAME

```
public static final java.lang.String ATTRIBUTE_NAME
```

**See Also:**: Constant Field Values

---


### BIPARTITION

```
public static final java.lang.String BIPARTITION
```

**See Also:**: Constant Field Values

---


### CONFIDENCE

```
public static final java.lang.String CONFIDENCE
```

**See Also:**: Constant Field Values

---


### RANKING

```
public static final java.lang.String RANKING
```

**See Also:**: Constant Field Values


**Constructor Detail**

### MulanPredict

```
public MulanPredict(int trainArffId,
                    int unlabeledArffId,
                    java.lang.String arffRecordsDatabase,
                    java.lang.String predictionsDirectory)
```


**Method Detail**

### generateInstancesNames

```
public java.util.Vector<java.lang.String> generateInstancesNames()
```

---


### getLogFile

```
public java.lang.String getLogFile()
```

---


### getTrainArff

```
public MulanArffRecord getTrainArff()
```

---


### getUnlabeledArff

```
public MulanArffRecord getUnlabeledArff()
```

---


### predict

```
public void predict()
```

---


### processOutput

```
public java.lang.String processOutput(int instanceIndex,
                                      mulan.classifier.MultiLabelOutput instanceOutput)
```

---


### savePredictions

```
public void savePredictions()
```

---


### setPredictionsFile

```
public void setPredictionsFile(java.lang.String predictionsDirectory)
```

---


### main

```
public static void main(java.lang.String[] args)
```

---


### predictionsHeader

```
public static java.lang.String predictionsHeader()
```


---


|  |  |  |
| --- | --- | --- |
| **Overview** \| **Package** \| **Class** \| **Use** \| **Tree** \| **Deprecated** \| **Index** \| **Help** | |  |
| PREV CLASS   **NEXT CLASS** | **FRAMES**    **NO FRAMES**     **All Classes** |  |
| SUMMARY: NESTED \| FIELD \| CONSTR \| METHOD | DETAIL: FIELD \| CONSTR \| METHOD |  |


---
